# Hybrid Compression Method for Trained 3D Gaussian Splatting Models Based on VQ and HEVC

**DOI:** 10.3390/s26134125

**Published:** 2026-06-30

**Authors:** Dong-Ha Kim, Byung-Yoon Choi, Kwan-Jung Oh, Gwangsoon Lee, Jae-Gon Kim

**Affiliations:** 1 Department of Smart Air Mobility, Korea Aerospace University, Goyang 10540, Republic of Korea; donghakim@kau.kr (D.-H.K.); bychoi@kau.kr (B.-Y.C.); 2 Electronics and Telecommunications Research Institute, Daejeon 34129, Republic of Korea; kjoh@etri.re.kr (K.-J.O.); gslee@etri.re.kr (G.L.)

**Keywords:** 3D Gaussian splatting, Gaussian splat coding, vector quantization, high efficiency video coding, parallel assignment linear sorting

## Abstract

3D Gaussian Splatting (3DGS) has recently emerged as an effective representation for immersive 3D scene rendering, providing high visual fidelity and real-time rendering efficiency. To support interoperable compression of trained 3DGS content, the Moving Picture Experts Group (MPEG) is exploring Gaussian Splat Coding (GSC), which mainly targets already trained 3DGS models following the INRIA reference format. The current video-based GSC anchor reorders 3DGS attributes into 2D attribute maps using Parallel Assignment Linear Sorting (PLAS) and compresses the resulting maps using High Efficiency Video Coding (HEVC). However, higher-order spherical harmonic coefficients (SH-AC) often remain irregular and exhibit low local spatial correlation even after PLAS reordering, limiting the coding efficiency of conventional video codecs. This paper proposes a VQ-HEVC hybrid compression framework that is structurally compatible with the video-based GSC anchor framework, in which SH-AC coefficients are represented by vector quantization (VQ) indices, while the remaining attributes are encoded using the same HEVC-based procedure as the GSC anchor. The proposed method adopts a two-stage VQ scheme that combines coarse VQ and product-quantization-based residual quantization, together with zero-masked residual VQ and flexible PQ grouping, to improve index-map coding efficiency across rate points. The generated VQ indices are packed into YUV400 index-map sequences and encoded using HEVC lossless coding, while the corresponding codebooks are transmitted as metadata. Experimental results on the Bartender and Cinema sequences of the MPEG GSC CTC demonstrate consistent rate–distortion improvements over the video-based GSC anchor across multiple objective quality metrics within the evaluated setting. In terms of RGB-PSNR, the proposed method achieves BD-rate reductions of 22.3% and 18.5% for the Bartender and Cinema datasets, respectively. These results suggest that, for the evaluated GSC CTC sequences, VQ-based SH-AC representation can effectively complement PLAS-based video coding while maintaining consistency with the existing GSC coding structure.

## 1. Introduction

Recently, 3D Gaussian Splatting (3DGS) has emerged as an effective representation for immersive 3D imaging and real-time novel-view synthesis [1]. A trained 3DGS model represents a scene as a set of anisotropic 3D Gaussians, each of which contains spatial and appearance-related attributes such as position, scale, rotation, opacity, and spherical harmonic (SH) coefficients. Owing to its explicit point-based structure, 3DGS enables high-fidelity rendering with real-time efficiency, making it suitable for immersive applications such as free-viewpoint video, virtual reality, and six-degrees-of-freedom (6DoF) media [2].

Despite these advantages, trained 3DGS models usually contain a large number of Gaussians, and each Gaussian is represented by dozens of floating-point attributes. This results in substantial storage and transmission costs, limiting the practical deployment of 3DGS in networked immersive media systems. To reduce model size, many studies have investigated pruning, quantization, attribute transformation, entropy modeling, and compact representation learning for 3DGS compression [3,4,5,6,7,8,9]. Among these approaches, vector quantization (VQ)-based and related quantization methods have also been explored because Gaussian attributes often exhibit statistical redundancy in the attribute space. For example, CompGS applies K-means-based VQ to compact Gaussian parameters, including SH coefficients [4]. Other compression methods, such as LightGaussian and MesonGS, also demonstrate the effectiveness of attribute-level compaction through pruning, VQ-related quantization, and attribute transformation [5,6]. However, from a standardization perspective, these methods are generally developed as standalone model-compression frameworks rather than as components of an interoperable coding pipeline. Therefore, their direct adoption into the video-based Gaussian Splat Coding (GSC) framework currently explored by the Moving Picture Experts Group (MPEG) is not straightforward.

From a standardization perspective, a key objective of 3DGS compression is to directly compress already trained 3DGS models without depending on a specific training method or alternative model structure. In practical applications, 3DGS content may be generated through various training pipelines and subsequently stored, transmitted, and distributed as trained models. Therefore, an interoperable compression framework should preserve the original attribute structure and rendering process of trained 3DGS models, rather than requiring retraining or conversion to a different representation.

Given the potential of 3DGS for immersive 3D video applications, MPEG has initiated standardization activities for Gaussian Splat Coding (GSC) to establish such an interoperable compression framework for trained 3DGS content [10,11,12]. These activities are conducted within the GSC Ad-Hoc Group (AhG), jointly operated by Working Group 7 (3D Graphics Coding) and Working Group 4 (Video Coding). To explore candidate technologies, Joint Exploration Experiment 6 (JEE 6) has been launched, defining experimental pipelines for both static and dynamic 3DGS compression. Within this effort, two main exploration tracks have been considered: I-3DGS, which targets models following the INRIA reference format, and A-3DGS (Alternative 3DGS) [13,14,15], which investigates alternative 3DGS model structures.

For static 3DGS compression, MPEG has explored extensions of existing point cloud compression standards, including Geometry-based Point Cloud Compression (G-PCC) [16] and Video-based Point Cloud Compression (V-PCC) [17], to facilitate faster standardization. Since trained 3DGS models have a point-based structure similar to point clouds, these extensions can exploit geometric proximity among 3D Gaussians to encode spatially coherent attributes while preserving the original model structure and rendering process.In parallel, a video-based GSC anchor has also been developed by extending conventional video coding tools [18]. This anchor first reorders 3DGS attributes into 2D attribute maps using Parallel Assignment Linear Sorting (PLAS) [19], and then compresses the resulting maps using High Efficiency Video Coding (HEVC) [20]. This design is attractive from a standardization perspective because it preserves the original 3DGS representation while reusing mature video coding technology. In this paper, we focus on this video-based GSC anchor and improve the compression efficiency of SH-AC coefficients within the same coding framework.

Nevertheless, the video-based GSC anchor has an inherent limitation when compressing higher-order SH coefficients, referred to as SH-AC coefficients. PLAS improves spatial correlation in the generated 2D attribute maps by reordering Gaussians based on their 3D positions and SH-DC coefficients, which represent the view-independent color components. However, SH-AC coefficients represent view-dependent appearance variations and are less directly correlated with spatial proximity. As a result, SH-AC attribute maps often remain irregular and exhibit relatively low local spatial correlation even after PLAS reordering, making them less suitable for direct compression by conventional 2D video codecs [20,21]. Since SH-AC coefficients account for a large portion of the 3DGS attribute dimensions and strongly influence view-dependent color fidelity, their inefficient compression can become a major bottleneck in the overall rate–distortion performance of the GSC pipeline.

To address this limitation, this paper presents a VQ-HEVC hybrid compression framework that is structurally compatible with the video-based GSC anchor framework. In the proposed method, SH-AC coefficients are represented by VQ indices, while the remaining attributes are encoded using the same HEVC-based procedure as the GSC anchor. Rather than introducing VQ as a standalone compression framework for 3DGS, this work focuses on integrating VQ-based SH-AC representation into the video-based GSC anchor framework. Specifically, a two-stage VQ structure combining coarse VQ and PQ-based residual quantization is adopted to reduce the quantization error of SH-AC coefficients. The resulting VQ indices are packed into 2D index maps and encoded as YUV400 video frames using HEVC lossless coding without modifying the HEVC encoder, decoder, or bitstream syntax. The corresponding VQ codebooks and auxiliary parameters are transmitted separately as metadata and are used in an inverse-VQ post-processing stage to reconstruct the SH-AC coefficients. In addition, zero-masked residual VQ is introduced to explicitly map small residual vectors to zero, thereby producing sparse residual index maps and improving their HEVC coding efficiency.

The main contributions of this paper are summarized as follows:

- An analysis of the spatial correlation characteristics of PLAS-reordered attribute maps identifies the inefficient compression of SH-AC coefficients as a major bottleneck of the PLAS-based GSC anchor.
- The proposed VQ-HEVC hybrid framework replaces SH-AC attribute maps with HEVC-coded VQ index maps while preserving structural compatibility with the video-based GSC anchor framework.
- The proposed two-stage VQ scheme combines coarse VQ and PQ-based residual quantization with a flexible PQ grouping strategy for efficient SH-AC representation.
- The proposed zero-masked residual VQ explicitly maps small residual vectors to the zero index, thereby increasing the sparsity of residual index maps and improving their HEVC coding efficiency.

Experimental results on the Bartender and Cinema sequences of the MPEG GSC CTC show that the proposed method achieves BD-rate reductions of 22.3%, 26.9%, and 20.1% in RGB-PSNR, YUV-PSNR, and SSIM for the Bartender dataset, respectively. For the Cinema dataset [22], BD-rate reductions of 18.5%, 20.2%, and 13.9% are obtained in the same metrics. These results indicate that VQ-based SH-AC representation can effectively complement PLAS-based video coding within the GSC framework.

The remainder of this paper is organized as follows. Section 2 reviews related studies on NeRF and 3DGS compression. Section 3 describes the structure of trained 3DGS models and the PLAS- and HEVC-based GSC anchor. Section 4 presents the proposed method, including two-stage VQ, zero-masked residual VQ, and HEVC-based index-map compression. Section 5 reports the experimental results. Finally, Section 6 concludes the paper and discusses future research directions.

## 2. Related Work

3D scene representation has evolved from implicit neural representations, such as NeRF, to explicit point-based representations, such as 3DGS. NeRF compression methods reduce the storage and computational costs of neural scene representations by compressing network weights, feature grids, or latent features using quantization, vector quantization (VQ), entropy coding, and low-rank factorization. Although these studies demonstrate that redundancy in neural scene representations can be effectively exploited, their techniques are not directly transferable to trained 3DGS models because NeRF does not use an explicit Gaussian attribute structure.

Compression methods for 3DGS generally combine pruning, scalar or vector quantization, attribute transformation, and entropy coding. LightGaussian reduces model size through importance-based pruning, SH degree reduction, and Gaussian vector quantization [5]. MesonGS applies post-training pruning, attribute transformation, region-adaptive hierarchical transform, and quantization, while representing SH coefficients using K-means-based VQ [6]. These approaches construct compact 3DGS representations by jointly modifying multiple Gaussian attributes and their coding procedures.

CompGS is one of the VQ-based methods most closely related to this work [4]. It applies K-means-based VQ to Gaussian parameters so that full-precision attributes are replaced by codebooks and indices. It also promotes zero opacity for redundant Gaussians, enabling pruning and reducing both model size and rendering complexity. Thus, VQ in CompGS is used as part of a compact representation for the overall 3DGS model.

Most existing 3DGS compression methods are designed as standalone frameworks and may involve pruning, finetuning, model-specific transformations, or independent entropy-coding structures. In contrast, this work does not replace the entire trained 3DGS representation. Instead, it targets the inefficient HEVC coding of SH-AC coefficients in the video-based MPEG GSC anchor. Only the SH-AC attribute maps are replaced with VQ index maps, while position, scale, rotation, opacity, and SH-DC coefficients follow the anchor procedure. Therefore, the contribution of this work lies not in the use of VQ itself, but in integrating VQ-based SH-AC representation into a structurally compatible VQ–HEVC hybrid framework for the video-based GSC anchor.

A direct numerical comparison with these standalone methods would not constitute a strictly controlled comparison because their reported results are obtained under different compression objectives, coding structures, and evaluation conditions. Existing methods may jointly modify multiple Gaussian attributes, prune Gaussians, reduce the SH degree, apply finetuning or rendering-based analysis, or use method-specific entropy-coding structures. In contrast, the present work preserves the anchor procedure for all attributes except SH-AC and evaluates the replacement of the SH-AC coding path under the MPEG GSC common test conditions. Accordingly, the video-based GSC anchor is used as the primary quantitative baseline to isolate the effect of the proposed SH-AC representation under consistent coding and evaluation conditions.

## 3. Background

Understanding the structure of a trained 3DGS model is essential for designing efficient compression methods. This section provides an overview of trained 3DGS attributes, PLAS-based attribute map generation, and the video-based GSC anchor.

### 3.1. Attributes of Trained 3DGS

A trained 3DGS model represents a 3D scene as a collection of structured 3D Gaussians characterized by spatial and appearance attributes. In this work, the 3DGS attributes follow the Gaussian Splatting model format, commonly referred to as the INRIA format [1]. Each Gaussian contains learnable parameters that are jointly optimized during training to minimize the photometric error between rendered images and the corresponding ground-truth (GT) multi-view images. During rendering, each Gaussian is projected onto the 2D image plane, where its spatial attributes determine the footprint and its appearance attributes control color and transparency.

Table 1 summarizes the attributes of each Gaussian. The spatial attributes consist of position, anisotropic scale, and orientation represented by a unit quaternion. For appearance representation, each Gaussian uses SH coefficients: the zeroth-order SH coefficients ($f_{\mathrm{dc}}$) represent RGB color components, while the higher-order SH coefficients ($f_{\mathrm{ac}}$) capture view-dependent color variations. In total, each Gaussian has 59 attributes.

### 3.2. PLAS for 3DGS Compression

PLAS improves compression efficiency by increasing spatial correlation within each 2D attribute grid through the reordering of Gaussian attribute vectors. This reordering is possible because rendering is invariant to the sequential ordering of Gaussians in the scene.

**Figure 1:**
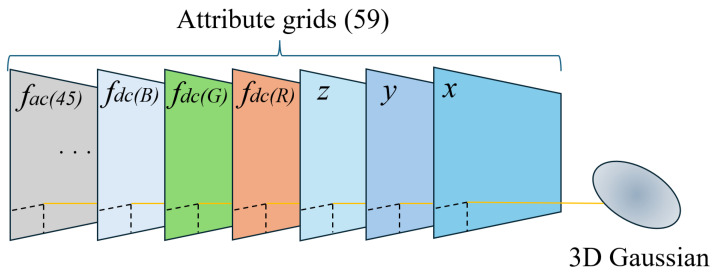
Mapping of each attribute of a 3D Gaussian to the corresponding 2D grid.

Figure 1 illustrates the mapping of Gaussian attributes onto 2D grids. Each Gaussian has 59 attributes, and each attribute is mapped to a separate grid, resulting in 59 attribute maps. These maps are spatially aligned so that all attributes of a given Gaussian occupy the same grid coordinate across different maps.

PLAS is based on Fast Linear Assignment Sorting (FLAS) [23]. In the sorting process, a six-dimensional vector composed of 3D position and SH-DC coefficients is used, based on the assumption that Gaussians with similar positions and colors are likely to share similar values in other attributes. Once the final permutation is obtained, it is uniformly applied to all attribute grids.

**Figure 2:**
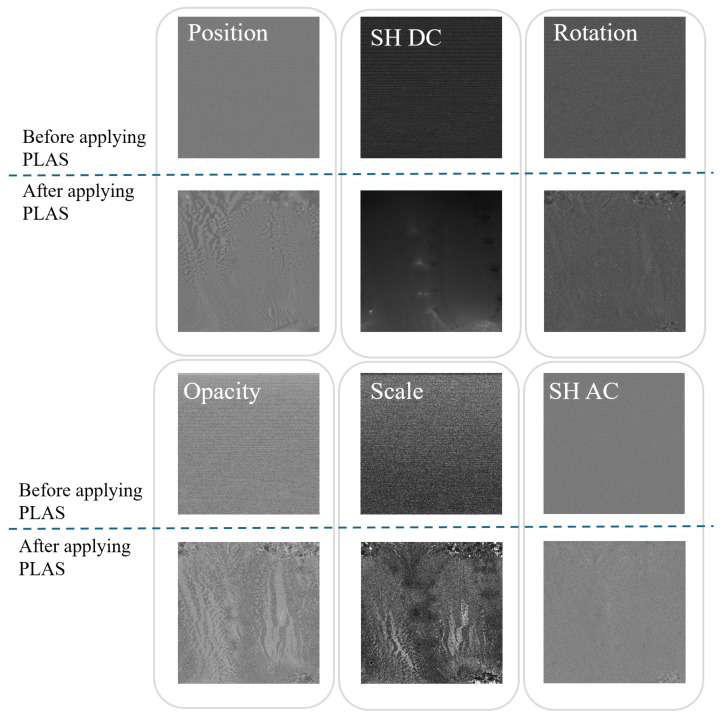
Comparison of trained 3DGS 2D attribute grids before and after PLAS-based reordering.

Figure 2 shows the attribute maps before and after PLAS-based reordering. Although the sorting relies only on 3D positions and SH-DC coefficients, most attributes exhibit improved spatial correlation after reordering, confirming that PLAS enhances the compression efficiency of trained 3DGS models.

### 3.3. Trained 3DGS Compression with HEVC

The GSC AhG has proposed the Test Model Candidate Video (TMCV) [18] as the implementation of the video-based GSC anchor for 3DGS compression. TMCV combines PLAS-based reordering with 2D video coding: PLAS first generates spatially correlated attribute maps, and the resulting maps are then compressed using HEVC. This anchor serves as a reference for evaluating and validating proposed technologies in the ongoing GSC standardization activity.

**Figure 3:**
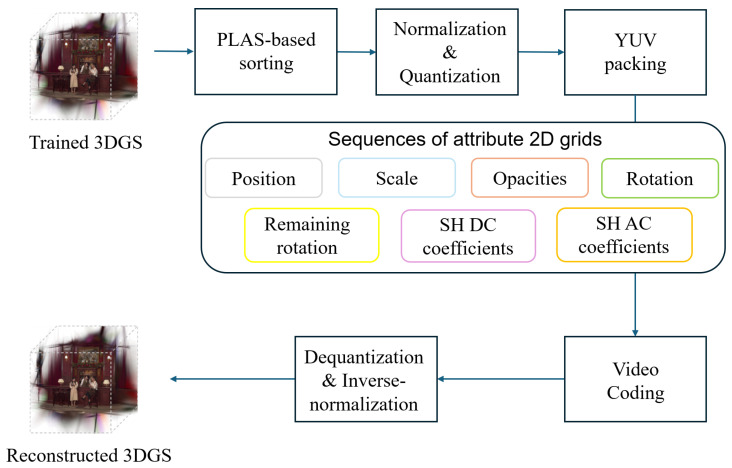
Pipeline of the video-based GSC anchor.

Figure 3 illustrates the pipeline of the video-based GSC anchor. PLAS reorders the Gaussians based on their position and SH-DC coefficients, and the resulting permutation is uniformly applied to all 59 attribute maps. Each attribute map is then packed into a video sequence and compressed using HEVC. The position attributes are quantized to 16 bits and encoded in HEVC lossless mode to preserve geometric accuracy. The remaining attributes are quantized to 10 bits after min-max normalization and packed into attribute-specific video sequences using either YUV444 or YUV400 format. As a result, all attributes of the trained 3DGS model are packed into seven video sequences and compressed using HEVC.

For the non-position attributes, Quantization Parameters (QPs) are assigned according to the relative importance of each attribute to support efficient bitrate allocation across different rate points. To reconstruct the 3DGS model, the compressed bitstreams are decoded, followed by dequantization and inverse normalization of the attribute maps. The reconstructed maps are then converted back into Gaussians for rendering.

## 4. Proposed Method

This section presents the proposed VQ-HEVC hybrid compression framework, which is structurally compatible with the video-based GSC anchor framework. The proposed method targets SH-AC coefficients, which remain weakly correlated in the 2D attribute maps even after PLAS-based reordering. The spatial correlation characteristics of PLAS-reordered attribute maps are first analyzed, followed by the proposed two-stage VQ, zero-masked residual VQ, and HEVC-based index-map coding.

Figure 2 compares the 2D attribute maps before and after PLAS-based reordering. As shown, position and SH-DC coefficients exhibit distinct spatial patterns after sorting, while scale, rotation, and opacity show moderate improvement in local spatial correlation. In contrast, the SH-AC coefficient maps remain spatially noisy, indicating that PLAS provides only limited improvement for these coefficients.

To quantify this observation, Table 2 reports the average neighbor correlation of each attribute map after PLAS-based reordering on the Bartender and Cinema datasets. For each frame, the neighbor correlation was computed as the average Pearson correlation coefficient between horizontally and vertically adjacent samples in each 2D attribute map, excluding boundary samples. The reported value for each attribute group was obtained by averaging the correlation values over all corresponding attribute dimensions and all frames in the sequence. As shown in the table, the SH-AC coefficient maps consistently show the lowest correlation values, confirming that their spatial structure remains highly irregular after PLAS and can limit the overall coding efficiency.

To address this limitation, the proposed method replaces the direct HEVC-based coding of SH-AC attribute maps with VQ-based representation. While the GSC anchor compresses each SH-AC attribute map sequence individually using HEVC, the proposed method clusters and quantizes SH-AC attribute vectors in the attribute space, exploiting similarity among SH-AC vectors for more efficient representation.

**Figure 4:**
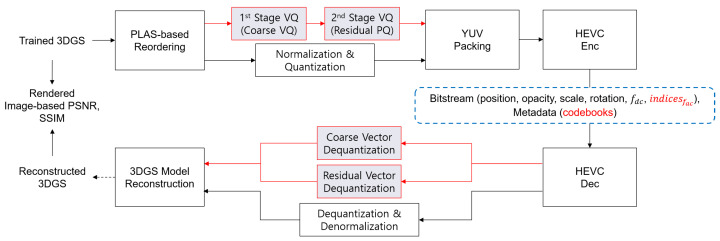
The proposed 3DGS compression pipeline, combining HEVC compression for spatially correlated attributes with VQ for SH-AC coefficients.

As illustrated in Figure 4, the proposed method is structurally integrated with the video-based GSC anchor framework. PLAS-based reordering is first applied following the GSC anchor. Then, VQ is applied to the SH-AC coefficients to generate VQ indices and codebooks, while the remaining attributes are encoded using the same HEVC-based procedure as the GSC anchor. The resulting VQ indices are packed into 2D index maps and compressed with HEVC, while the VQ codebooks and the associated parameters are transmitted as metadata. At the decoder, the SH-AC coefficients are reconstructed from the decoded index maps and the transmitted metadata through inverse-VQ post-processing.

Directly applying a single-stage VQ to the entire 45-dimensional SH-AC vector can lead to large quantization errors. To address this, a two-stage VQ scheme combining Residual Vector Quantization (RVQ) [24] and Product Quantization (PQ) [25] is employed. In addition, zero-masked residual VQ is introduced to explicitly map small residual vectors to the zero index, thereby increasing the sparsity of residual index maps and improving the HEVC coding efficiency of the index maps.

### 4.1. Two-Stage VQ

**Figure 5:**
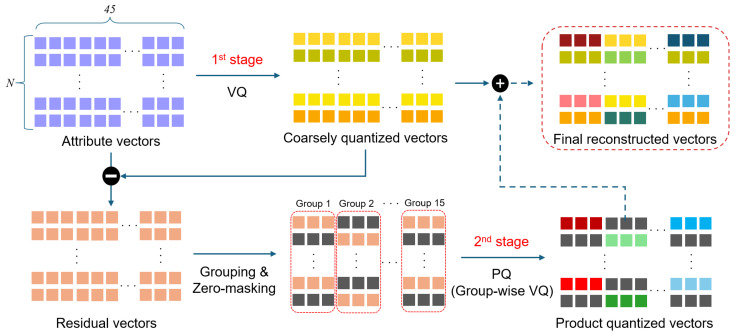
Overview of the proposed two-stage VQ process. The first stage performs coarse VQ on SH-AC coefficient vectors, and the second stage applies PQ-based residual quantization using KNN-based sub-vector grouping. The final reconstructed vectors are obtained by adding the PQ-quantized residuals to the coarsely quantized vectors from the first stage.

Figure 5 illustrates the proposed two-stage VQ process. Before quantization, the SH-AC coefficient vector of each Gaussian is normalized independently for each attribute dimension. In the first stage, VQ is applied to the full 45-dimensional SH-AC vector to generate a coarse quantized vector. In the second stage, the residual vector, defined as the difference between the original SH-AC vector and the first-stage reconstructed vector, is further quantized to reduce the remaining quantization error. To improve residual quantization performance, a K-Nearest Neighbors (KNN)-based greedy grouping method is used to organize statistically similar residual attribute dimensions into nonoverlapping sub-vectors, and PQ is performed independently on each sub-vector. The grouping feature, distance metric, and assignment procedure are described below.

Before codebook training, the 45 SH-AC coefficient dimensions are independently normalized over all Gaussians in the input model. Let $x_{i,d}$ denote the coefficient of the *d*-th SH-AC dimension for the *i*-th Gaussian. The normalized coefficient is given by

(1) $${\tilde{x}}_{i,d}=\frac{x_{i,d}-x_{d}^{\min}}{\max\left(x_{d}^{\max}-x_{d}^{\min},\epsilon \right)},$$

where $x_{d}^{\min}$ and $x_{d}^{\max}$ are the minimum and maximum values of the *d*-th dimension over all Gaussians, respectively, and $\epsilon =10^{-8}$ prevents numerical instability. The per-dimension minimum and maximum values are retained as metadata, and the normalization span is calculated from them at the decoder.

The VQ codebooks are trained using MiniBatch K-means, which updates the codewords using subsets of the input vectors. For centroid initialization, the first center is selected randomly, and each subsequent center is sampled with probability proportional to its squared distance from the nearest selected center. The first-stage codebook is trained on the full 45-dimensional SH-AC vectors, whereas the second-stage codebooks are trained independently on the residual sub-vectors obtained by KNN-based grouping. The codebooks and groupings are generated separately for each compressed 3DGS model.

The KNN-based grouping is performed on the residual coefficients obtained after the first-stage VQ rather than on the original SH-AC coefficients. Let

(2) $$r_{d}={\left[r_{1,d},r_{2,d},\ldots ,r_{N,d}\right]}^{T}$$

denote the residual vector of the *d*-th SH-AC dimension across *N* Gaussians. To compare residual patterns across dimensions, each dimension-wise residual vector is independently min–max normalized as

(3) $${\bar{r}}_{i,d}=\frac{r_{i,d}-r_{d}^{\min}}{\max\left(r_{d}^{\max}-r_{d}^{\min},\epsilon \right)},$$

where $r_{d}^{\min}$ and $r_{d}^{\max}$ are the minimum and maximum residual values of the *d*-th dimension over all Gaussians, respectively. The normalized residual-dimension vector is then defined as

(4) $${\bar{r}}_{d}={\left[{\bar{r}}_{1,d},{\bar{r}}_{2,d},\ldots ,{\bar{r}}_{N,d}\right]}^{T}.$$

The distance between dimensions *d* and *j* is measured using the Euclidean distance:

(5) $$\delta \left(d,j\right)={∥{\bar{r}}_{d}-{\bar{r}}_{j}∥}_{2}.$$

Using the pairwise distances among the 45 residual dimensions, an unassigned dimension is selected as the reference dimension and greedily grouped with its nearest unassigned dimensions until the required group size is reached. This produces nonoverlapping groups in which every SH-AC dimension is assigned exactly once. For *G* groups, each sub-vector contains 45/G dimensions; therefore, configurations with one, three, five, and nine groups use sub-vector dimensions of 45, 15, 9, and 5, respectively.

A codebook size of 1024 is used in both stages; therefore, each SH-AC vector is represented by one 10-bit coarse index and one 10-bit residual index per sub-vector group. The SH-AC bitrate is controlled by varying the number of residual groups. The residual representation also supports the zero-masking scheme described in the following subsection.

### 4.2. Zero-Masked Residual VQ

The coding efficiency of HEVC-coded index maps depends heavily on the distribution of index values. Zero-masked residual VQ maps small-magnitude residual sub-vectors to the zero index on a group-wise basis, increasing index-map sparsity and reducing entropy.

**Figure 6:**
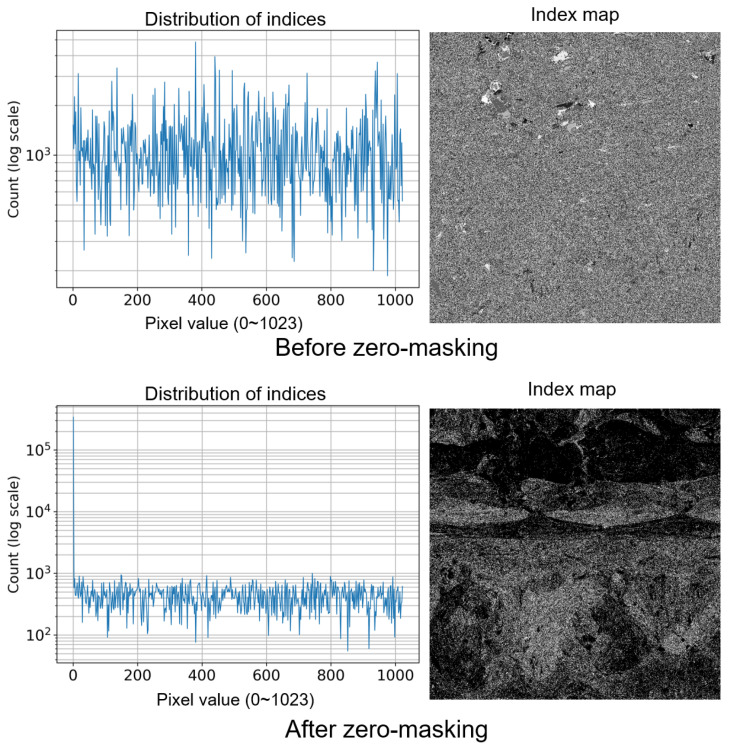
An example of zero-masking: distribution of VQ indices shown in log scale and the corresponding index map.

Figure 6 compares the residual VQ index distribution before and after zero-masking and shows example index maps. After zero-masking, the occurrence of zero indices increases, and the zero-valued regions in the index map become more prominent. The zero-masking threshold is defined as the percentage of residual vectors to be masked and controls the trade-off between bitrate and quantization error. A higher threshold maps a larger number of residual vectors to the zero index, which reduces the number of nonzero indices and lowers the bitrate, but increases quantization error.

### 4.3. HEVC-Based Encoding of VQ Indices

In the proposed method, the corresponding VQ indices are arranged into 2D index maps and encoded using HEVC, whereas the anchor directly encodes SH-AC attribute maps.

The 10-bit indices generated from the first-stage coarse VQ are packed into a single frame, while the VQ indices from each second-stage sub-vector group are represented as individual frames. Thus, the total number of index frames is equal to the number of sub-vector groups plus one coarse frame. Since each VQ index is a single-channel scalar value, the index frames are arranged as a YUV400 video sequence. The sequence is encoded using HEVC in lossless mode to preserve the VQ indices exactly, because any change in an index may select a different codeword during reconstruction.

Zero-masking increases the sparsity of the residual index maps, improving their HEVC lossless coding efficiency. At the decoder, the index bitstream is decoded using an unmodified HEVC decoder. The decoded indices are used to retrieve the corresponding coarse and residual codewords from the transmitted codebooks. The residual codewords are reordered using the transmitted grouping information, added to the coarse codeword, and inverse-normalized to reconstruct the 45-dimensional SH-AC vector. The reconstructed SH-AC coefficients are then combined with the remaining decoded attributes to generate a trained 3DGS model in the format accepted by the official MPEG-GSC renderer.

## 5. Experimental Results

In the GSC Common Test Conditions (CTC), two datasets, Bartender and Cinema [22], are defined for JEE 6. Each dataset consists of 20 Full HD multi-view images and provides a trained 3DGS model for compression experiments. In GSC, compression performance is evaluated by comparing the rendered images of the original trained 3DGS model with those of the reconstructed model after compression. Thus, the rendered images from the original trained 3DGS model are used as the reference, rather than the original multi-view images used for training. Rendering is performed using the official MPEG-GSC renderer, and quality is measured using RGB-PSNR, YUV-PSNR, and SSIM together with the compressed model size. For a fair comparison with the GSC anchor, the proposed method is evaluated at rate points (RPs) corresponding to similar target bitrates. The present evaluation is restricted to the Bartender and Cinema sequences specified in the GSC CTC considered in this study. Accordingly, the reported results demonstrate the effectiveness of the proposed method within this experimental setting, while further evaluation on more diverse scene categories is needed to assess broader generalization.

### 5.1. Anchor for GSC

As described in Section 3.3, the GSC anchor first reorders the attributes of a trained 3DGS model using PLAS, maps the reordered attributes onto 2D grids, and compresses the resulting attribute map sequences using HEVC. In CTC, the HEVC Test Model (HM) version 18.0 is used as the video codec. Each attribute video is independently compressed with QPs assigned according to attribute importance, as summarized in Table 3.

The position attribute is compressed using HEVC lossless mode because even small geometric errors can significantly degrade rendering quality. For the remaining attributes, predefined QP values are used to support multiple bitrate operating points.

### 5.2. VQ Codebook Training and KNN Grouping Settings

All VQ codebooks were trained using MiniBatch K-means with a single initialization run, a maximum of 30 iterations, and a mini-batch size of 4096. Training was terminated when no sufficient decrease in the K-means objective was observed over 10 consecutive mini-batches or when the maximum number of iterations was reached.

Fixed random seeds were used for reproducibility. The random seed was initialized to 0 for the first-stage coarse VQ and incremented sequentially for the residual sub-vector groups. A separate random seed of 0 was used to determine the order in which reference dimensions were selected during the KNN-based grouping procedure. The codebooks and KNN-based groupings were generated independently for each compressed 3DGS model.

The codebook size was fixed to 1024 for both stages in all experiments; therefore, each coarse and residual VQ index was represented using 10 bits.

As summarized in Table 4, the metadata primarily consists of the coarse and residual VQ codebooks. The coarse codebook contains 1024×45 Float32 coefficients. For *G* residual groups, the residual codebooks collectively contain G×1024×(45/G) Float32 coefficients; therefore, its total raw size is independent of *G*. The remaining metadata include the per-dimension SH-AC minimum and maximum values and an ordered list of 45 grouping indices. The total fixed raw metadata payload is 0.369045 MB per compressed 3DGS model.

In the experimental implementation, the codebooks, normalization parameters, grouping information, VQ configuration, and decoder parameters were stored in a separate uncompressed NPZ file. For the rate–distortion evaluation, the actual serialized NPZ file size, including the NPZ container overhead and auxiliary parameters, was included in the total compressed model size.

### 5.3. Fixed Sub-Vector Group Configuration

As illustrated in Figure 4, the proposed method applies VQ to SH-AC coefficients and compresses the generated indices using HEVC, while the remaining attributes are encoded in the same manner as the GSC anchor. In the fixed configuration, the proposed two-stage VQ is configured as follows:

1. Stage 1 (Coarse VQ): The 45-dimensional SH-AC vector is coarsely quantized using a 1024-entry codebook, producing one 10-bit index.
2. Stage 2 (Residual PQ): The residual vector is divided into nine 5-dimensional sub-vector groups, each quantized using a 1024-entry codebook and represented by a 10-bit index.

Thus, each Gaussian is represented by one coarse index and nine residual indices. The resulting ten index maps are encoded as a YUV400 sequence using HEVC lossless mode, with the codebooks, normalization parameters, and grouping information conveyed as metadata.

For the remaining attributes, the QPs listed in Table 3 are applied at each RP, following the GSC anchor. In the proposed method, the zero-masking threshold controls the bitrate by determining the percentage of residual vectors mapped to zero. A higher threshold maps more residual vectors to zero, which reduces the number of nonzero residual indices and lowers the bitrate, but increases reconstruction error.

To illustrate the rate–quality trade-off controlled by zero-masking, Table 5 reports results for multiple thresholds at RP3 on both the Cinema and Bartender datasets. The RP-dependent threshold schedule was selected based primarily on preliminary rate–distortion experiments conducted on Cinema. The same threshold values were then evaluated on Bartender without sequence-specific retuning. For both datasets, increasing the threshold consistently reduces the compressed model size at the cost of lower RGB-PSNR. Although the exact rate–distortion values are scene-dependent, Bartender exhibits a trend similar to that observed for Cinema. Based on these preliminary experiments, thresholds of 0.95, 0.8, 0.6, and 0 were empirically selected for RP1–RP4, respectively. Stronger masking is used at lower-rate points, whereas masking is progressively relaxed as the target quality increases and disabled at RP4.

**Figure 7:**
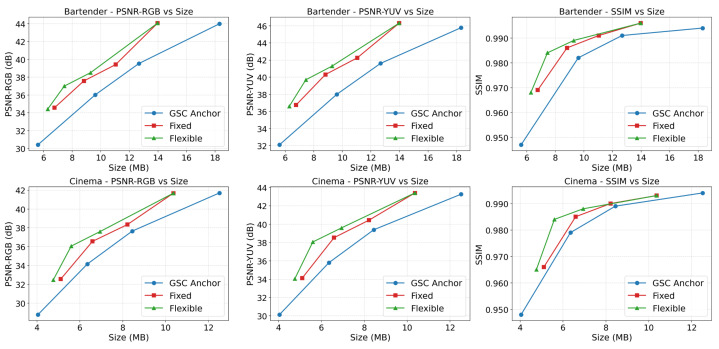
Rate–distortion curves of the GSC anchor and the proposed method on the Bartender and Cinema datasets.

Table 6 compares the compression performance of the GSC anchor and the proposed method with the fixed sub-vector configuration. The model size includes the HEVC bitstream and all transmitted metadata. For both datasets, the proposed method generally achieves comparable or better rendering quality with reduced model size, especially at high-quality RPs. The corresponding RD curves are shown in Figure 7.

As shown in Table 7, the metadata size remains approximately 0.37 MB for all RPs and both datasets because the same codebook dimensions, numerical precision, and grouping representation are used, although the codebook values are trained independently for each model.

The HEVC-coded SH-AC index-map bitstream increases from RP1 to RP4 as the zero-masking threshold is progressively reduced. Although the spatial dimensions and number of samples in the index maps remain unchanged, a higher zero-masking threshold maps more residual indices to zero, resulting in a more concentrated index distribution and improving the entropy-coding efficiency of HEVC lossless coding. Consequently, RP1 yields the smallest coded index-map bitstream, whereas RP4, which applies no zero-masking, results in the largest one. This trend is consistently observed for both datasets. As summarized in Table 8, the proposed SH-AC representation provides substantial BD-rate reductions even after all metadata overhead is included.

These results confirm that selectively applying VQ to SH-AC coefficients, which exhibit low spatial correlation after PLAS, is effective for improving compression efficiency.

### 5.4. Flexible Sub-Vector Group Configuration

As described in Section 4.1, to improve rate–distortion performance, the number of residual sub-vector groups is adjusted for each RP while the codebook size remains fixed. The zero-masking ratio and group configuration are listed in Table 9.

Table 10 presents the results under the flexible sub-vector group configuration. Compared with the fixed nine-group configuration, adjusting the number of residual sub-vector groups at each RP improves the balance between index bitrate and reconstruction quality.

Table 11 shows that using fewer residual groups reduces the SH-AC index-map bitstream at RP1–RP3, while the remaining-attribute bitstreams and metadata remain unchanged. At RP4, both configurations use nine groups without zero-masking and therefore produce identical sizes. Flexible grouping provides approximately 5–6% additional BD-rate reductions, as summarized in Table 8.

### 5.5. Ablation Study

**Figure 8:**
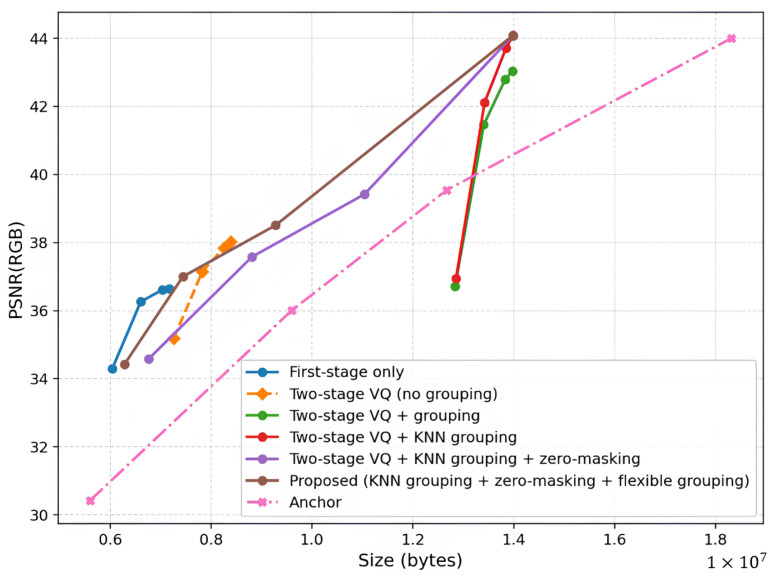
RD curves of the ablation study on the Bartender dataset. The first-stage-only configuration covers a limited low-quality range, whereas the residual VQ-based configurations extend the achievable quality to the high-quality operating points of the anchor.

To evaluate the contribution of each component, an ablation study was conducted on the Bartender dataset. The RD curves and BD-rate results are shown in Figure 8 and Table 12, respectively.

The RGB-PSNR ranges in Table 12 indicate the quality coverage of the evaluated rate points for each configuration. Each BD-rate was calculated over the overlapping RGB-PSNR interval between the corresponding configuration and the anchor. Because the achievable quality ranges differ substantially among the configurations, the BD-rate values should be interpreted together with their corresponding quality coverage rather than as a direct ranking over an identical quality interval. Accordingly, the following component-level observations are discussed as indicative trends under the evaluated operating ranges, rather than as a strict numerical ranking of the individual components.

The first-stage-only configuration shows a large BD-rate reduction of 30.9% over its overlapping interval with the anchor, mainly because each SH-AC vector is represented using only one 10-bit coarse index. However, it covers only 34.3–36.7 dB. Without residual information, the remaining coarse-VQ error imposes a reconstruction-quality ceiling, preventing this configuration from reaching the high-quality operating points of the anchor. Therefore, this BD-rate value reflects the rate-saving tendency in a limited low-quality interval rather than superior performance over the full quality range.

Adding residual VQ reduces the remaining SH-AC quantization error and extends the achievable quality range. Full-vector residual VQ reaches approximately 38.1 dB, whereas PQ-based grouped configurations extend the maximum RGB-PSNR to approximately 43–44 dB. This extension requires additional residual indices and group-specific codebooks, increasing the residual-stage bitrate. Thus, the residual stage is necessary to overcome the quality ceiling of coarse VQ and support high-quality operating points.

Naive nine-group PQ shows a 1.0% BD-rate increase over its overlapping interval, mainly because the additional residual index maps and statistically unrelated dimension groupings offset the reduction in quantization error. With KNN grouping, the observed BD-rate trend changes to −3.5%, suggesting that grouping dimensions with similar residual patterns is beneficial. With zero-masking, the observed BD-rate trend changes to −17.2%, indicating that increased residual index-map sparsity contributes to rate reduction. The flexible grouping configuration shows a BD-rate value of −22.3% while covering approximately 34.4–44.1 dB, suggesting that reducing the number of residual index maps at lower-rate points can improve the overall rate–distortion trade-off within the evaluated range.

### 5.6. Comparison of Complexity

The GSC anchor encodes a fixed number of SH-AC attribute frames using HEVC prediction and quantization, whereas the proposed method losslessly encodes a variable number of VQ index frames depending on the RP. Specifically, the proposed method generates two index frames at RP1 and ten at RP4 because more residual sub-vector groups are used at higher RPs.

Table 13 compares the encoding and decoding times of the GSC anchor and the proposed method on the Bartender and Cinema datasets. Each result was averaged over five runs. Despite including codebook training and VQ-index generation, the proposed method achieves lower total encoding time at all RPs for both datasets.

The decoding time of the proposed method remains relatively stable across the RPs, while that of the anchor decreases at lower RPs. Therefore, the proposed method is slightly slower at Bartender RP1 because the anchor processes a simpler SH-AC bitstream, whereas the inverse-VQ reconstruction cost changes little across the RPs. At the remaining RPs, the proposed decoding time is comparable to or lower than that of the anchor.

Peak memory usage was measured from a representative complete execution at Bartender RP4. The GSC anchor and the proposed method required approximately 2.31 GiB and 2.15 GiB, respectively, corresponding to a 7.1% reduction for the proposed method.

All complexity measurements were conducted on an Intel Core i9-14900K workstation with 62 GiB of memory running Ubuntu 20.04.6 LTS, without GPU acceleration. HEVC encoding and decoding used HM 18.0 with Range Extensions, compiled for 64-bit Linux using GCC 9.4.0. Each timing result was averaged over five runs. The reported VQ encoding time includes codebook training, VQ-index generation, and metadata serialization, while the VQ decoding time includes inverse-VQ reconstruction.

### 5.7. Comparison of Rendering Quality

For a fair visual comparison, the GSC anchor and the proposed method were evaluated using the same QP settings for all attributes except SH-AC, so that the observed differences mainly reflect the effect of the SH-AC compression method. The comparison was therefore conducted at RP2 on the Bartender dataset. Under this condition, the proposed method produces a bitstream size of 7.44 MB, which is smaller than the 9.60 MB produced by the anchor.

**Figure 9:**
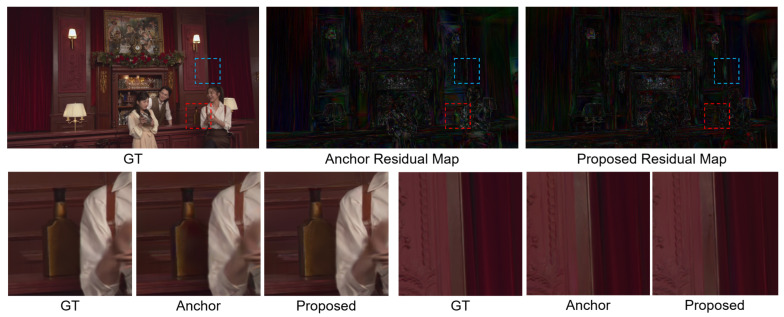
Rendering quality comparison on the Bartender dataset for View 6. The top row shows the rendering of the uncompressed input 3DGS model as the ground-truth reference (GT), followed by the residual maps of the anchor and the proposed method with respect to the GT. The bottom row shows enlarged rendered crops of the GT, anchor, and proposed method for the red- and blue-boxed regions. The red box indicates a region where the proposed method produces a smaller residual than the anchor, whereas the blue box indicates a region where the anchor produces a smaller residual than the proposed method.

Figure 9 presents a rendering-quality comparison for View 6 of the Bartender dataset. In the top row, the rendering of the uncompressed input 3DGS model is used as the ground-truth reference (GT), together with the residual maps computed between the GT and the rendered results of the anchor and the proposed method, respectively. In the bottom row, the actual rendered results of the GT, anchor, and proposed method are enlarged for the red- and blue-boxed regions in which the two residual maps exhibit noticeable differences. Since the primary difference between the two methods lies in the compression of the SH-AC coefficients, the comparison focuses on color fidelity and tonal consistency. The residual maps were generated using a common linear scale by multiplying the absolute RGB difference by 5 and clipping it to [0,255], without image-wise min–max normalization. Thus, identical intensities indicate identical residual magnitudes. Black denotes negligible error, red moderate error, and yellow or white relatively large error. The red and blue boxes indicate regions where the proposed method and the anchor, respectively, produce smaller residuals.

In the red-boxed region, neither the anchor nor the proposed method produces noticeable visual noise or structural artifacts. However, the enlarged rendered results show that the proposed method reproduces the color tones of the bottle and the surrounding wall more closely to the GT. Accordingly, the proposed method produces a smaller residual than the anchor in this region. This result shows that the proposed method can provide higher color fidelity in some local regions while operating with a smaller compressed size.

In contrast, in the blue-boxed region, the anchor maintains the colors and tones of the GT relatively consistently, whereas some Gaussians reconstructed by the proposed method exhibit colors that are inconsistent with the surrounding region. This results in a localized color inconsistency near the boundary between the curtain and the wall, where several pixels appear visually separated from their surroundings. Consequently, the proposed method produces a larger residual than the anchor in this region.

These differences can be attributed to the different ways in which the two methods exploit correlation among the SH-AC coefficients. In the anchor, PLAS arranges the SH-AC coefficients of spatially neighboring Gaussians at adjacent positions in the 2D attribute maps, allowing HEVC spatial prediction to exploit local correlation among neighboring samples during compression and reconstruction. Consequently, relatively consistent color characteristics can be maintained among adjacent Gaussians. In contrast, the proposed method independently quantizes the SH-AC vector of each Gaussian by mapping it to a VQ codevector. Although PLAS places neighboring Gaussians close to one another in the attribute maps, the VQ codevector-selection process does not directly use the reconstructed values or spatial predictions of neighboring Gaussians. Therefore, a codevector whose color characteristics differ from those of the surrounding region may occasionally be assigned to an individual Gaussian, resulting in localized color inconsistency.

Overall, the proposed method maintains rendering quality comparable to that of the anchor at a smaller bitstream size and reproduces colors closer to the GT in some regions. However, because VQ does not directly exploit spatial correlation among neighboring Gaussians, localized color distortions that are inconsistent with the surrounding tones may occur in certain regions.

**Figure 10:**
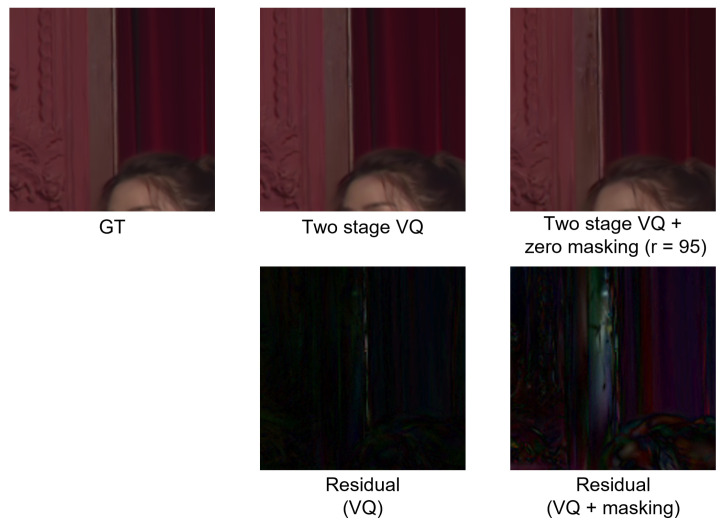
Visual comparison of the proposed two-stage VQ method without and with zero-masking on the Bartender dataset for View 6. The comparison is performed before HEVC video coding and shows only the VQ-based reconstruction results. The second stage uses nine residual sub-vector groups, and the zero-masking ratio is set to 0.95. The top row shows the rendered images, while the bottom row presents the corresponding residual maps with respect to the reference rendering.

Figure 10 compares the proposed two-stage VQ method without and with zero-masking on the Bartender dataset for View 6. This comparison is performed before HEVC video coding, using only the VQ-based reconstruction results, in order to isolate the visual effect of zero-masking. The second stage uses nine residual sub-vector groups, and the zero-masking ratio is set to 0.95. Applying zero-masking increases localized errors in some regions, but the overall residual magnitude remains comparable to that without zero-masking. This indicates that zero-masking can reduce bitrate while largely preserving rendering quality, although localized color distortions may occur.

## 6. Conclusions

In this paper, a VQ-HEVC hybrid compression framework that is structurally compatible with the video-based GSC anchor framework was proposed for trained 3DGS models. The proposed method targets SH-AC coefficients, whose spatial correlation remains low even after PLAS reordering in the GSC anchor. To address this limitation, SH-AC coefficients are represented by VQ indices using a two-stage VQ scheme consisting of coarse VQ and residual PQ, while the remaining attributes are encoded using the same HEVC-based procedure as the anchor. Zero-masked residual VQ further improves the coding efficiency of VQ indices by explicitly mapping small residual vectors to the zero index, thereby increasing the sparsity of residual index maps. The generated VQ indices are packed into index-map sequences and compressed using the unmodified HEVC coding path, while the reconstructed SH-AC coefficients are converted back into the original trained 3DGS format for rendering with the unmodified MPEG-GSC renderer.

Experimental results on the Bartender and Cinema sequences of the MPEG GSC CTC showed that the proposed method provides substantial BD-rate reductions compared with the GSC anchor within the evaluated setting. With the flexible sub-vector group configuration, BD-rate reductions of 22.3%, 26.9%, and 20.1% were obtained in RGB-PSNR, YUV-PSNR, and SSIM for the Bartender dataset, and 18.5%, 20.2%, and 13.9% for the Cinema dataset, respectively. Visual comparisons also showed that the proposed method maintains rendering quality comparable to the anchor at lower bitrates, although local color distortions may occur in some regions due to SH-AC quantization.

These results indicate that, within the evaluated GSC CTC setting, VQ-based SH-AC representation can effectively complement PLAS-based video coding within the GSC framework. The proposed method preserves structural and experimental compatibility with the video-based GSC anchor by retaining its PLAS preprocessing, HEVC-based map coding, reconstructed 3DGS format, and official rendering process. However, because the current implementation uses a separate NPZ file for VQ metadata and requires inverse-VQ post-processing after HEVC decoding, bitstream-level conformance to a candidate GSC syntax is not claimed.

The proposed approach may also be extended to tracked dynamic 3DGS sequences in GSC, where temporal correspondence between Gaussians may support reference-based inter-frame VQ of attribute residuals. However, temporal variations may reduce the effectiveness of fixed references and codebooks. Future work will investigate adaptive inter-frame VQ together with standardized signaling and parsing of VQ metadata.

## Figures and Tables

**Table 1:** Attributes of a single 3D Gaussian in a trained 3DGS model.

Symbol	Attribute	Number of Attributes
x,y,z	Position	3
$s_{x},s_{y},s_{z}$	Scale	3
$w,q_{x},q_{y},q_{z}$	Rotation	4
α	Opacity	1
$f_{\mathrm{dc}}$	SH-DC coefficients	3
$f_{\mathrm{ac}}$	SH-AC coefficients	45

**Table 2:** Average neighbor correlation of each attribute map after PLAS-based reordering.

Attribute	Bartender	Cinema
Position (x,y,z)	0.6591	0.8579
Scale ($s_{x},s_{y},s_{z}$)	0.5583	0.4752
Rotation ($w,q_{x},q_{y},q_{z}$)	0.5219	0.4733
Opacity (α)	0.4614	0.4707
SH-DC coefficients ($f_{\mathrm{dc}}$)	0.5871	0.6582
SH-AC coefficients ($f_{\mathrm{ac}}$)	0.3161	0.3364

**Table 3:** QP settings for attribute-wise video compression in the GSC anchor.

RP	Pos	Opa	Rot	Scl	SH-DC	SH-AC
1	Loss-less	22	17	12	7	37
2	Loss-less	17	7	12	7	27
3	Loss-less	17	2	7	7	22
4	Loss-less	7	2	7	7	12

**Table 4:** Metadata overhead of the proposed VQ representation. The listed sizes denote the fixed raw payload of each metadata component.

Metadata Component	Representation	Precision	Raw Size (MB)
Coarse VQ codebook	1024×45	Float32	0.18432
Residual PQ codebooks	G×1024×(45/G)	Float32	0.18432
Normalization parameters	2×45	Float32	0.00036
Sub-vector grouping	45 ordered dimension indices	UInt8	0.000045
**Total fixed raw payload**	–	–	**0.369045**

**Table 5:** Impact of the zero-masking threshold on compressed model size and RGB-PSNR for the Bartender and Cinema datasets at RP3.

Threshold	Bartender		Cinema	
	Size (MB)	RGB-PSNR (dB)	Size (MB)	RGB-PSNR (dB)
0	13.84	43.71	10.25	41.40
0.2	13.50	42.68	10.01	40.91
0.4	12.49	40.95	9.26	39.72
0.6	11.04	39.42	8.22	38.35
0.8	9.24	38.04	6.90	37.18
0.95	7.75	37.13	5.83	36.32

**Table 6:** Performance comparison between the GSC anchor and the proposed method with a fixed sub-vector configuration.

RP	Bartender								Cinema							
	GSC Anchor				Proposed Method				GSC Anchor				Proposed Method			
	RGB PSNR	YUV PSNR	SSIM	Size (MB)	RGB PSNR	YUV PSNR	SSIM	Size (MB)	RGB PSNR	YUV PSNR	SSIM	Size (MB)	RGB PSNR	YUV PSNR	SSIM	Size (MB)
1	30.42	32.12	0.947	5.60	34.58	36.76	0.969	6.76	28.78	30.14	0.948	4.05	32.55	34.13	0.966	5.11
2	36.01	37.99	0.982	9.60	37.58	40.31	0.986	8.81	34.15	35.80	0.979	6.35	36.56	38.53	0.985	6.59
3	39.54	41.61	0.991	12.67	39.42	42.26	0.991	11.04	37.65	39.39	0.989	8.44	38.35	40.44	0.990	8.22
4	44.00	45.78	0.994	18.31	44.08	46.32	0.996	13.98	41.71	43.27	0.994	12.51	41.68	43.41	0.993	10.36

**Table 7:** Compressed model size breakdown of the proposed method with the fixed sub-vector configuration.

Dataset	RP	Remaining Attributes (MB)	SH-AC Index Maps (MB)	Metadata (MB)	Total Size (MB)
Bartender	1	5.15	1.24	0.37	6.76
	2	5.72	2.72	0.37	8.81
	3	6.14	4.53	0.37	11.04
	4	6.28	7.33	0.37	13.98
Cinema	1	3.84	0.90	0.37	5.11
	2	4.25	1.97	0.37	6.59
	3	4.58	3.27	0.37	8.22
	4	4.67	5.32	0.37	10.36

**Table 8:** BD-rate performance of the two sub-vector configurations.

Sub-Vector Group	Dataset	RGB-PSNR	YUV-PSNR	SSIM
Fixed 9 groups	Bartender	−17.1%	−21.4%	−14.2%
	Cinema	−12.1%	−13.9%	−6.8%
Flexible groups	Bartender	−22.3%	−26.9%	−20.1%
	Cinema	−18.5%	−20.2%	−13.9%

**Table 9:** Flexible sub-vector group configuration per rate point.

RP	Zero-Masking Ratio	Number of Sub-Vector Groups
1	0.95	1
2	0.8	3
3	0.6	5
4	0	9

**Table 10:** Performance comparison between the GSC anchor and the proposed method with a flexible sub-vector configuration.

RP	Bartender								Cinema							
	GSC Anchor				Proposed Method				GSC Anchor				Proposed Method			
	RGB PSNR	YUV PSNR	SSIM	Size (MB)	RGB PSNR	YUV PSNR	SSIM	Size (MB)	RGB PSNR	YUV PSNR	SSIM	Size (MB)	RGB PSNR	YUV PSNR	SSIM	Size (MB)
1	30.42	32.12	0.947	5.60	34.42	36.60	0.968	6.28	28.78	30.14	0.948	4.05	32.46	34.04	0.965	4.76
2	36.01	37.99	0.982	9.60	37.00	39.70	0.984	7.44	34.15	35.80	0.979	6.35	36.06	38.06	0.984	5.60
3	39.54	41.61	0.991	12.67	38.51	41.30	0.989	9.28	37.65	39.39	0.989	8.44	37.60	39.60	0.988	6.94
4	44.00	45.78	0.994	18.31	44.08	46.32	0.996	13.98	41.71	43.27	0.994	12.51	41.68	43.41	0.993	10.36

**Table 11:** Compressed model size breakdown of the proposed method with the flexible sub-vector configuration.

Dataset	RP	Remaining Attributes (MB)	SH-AC Index Maps (MB)	Metadata (MB)	Total Size (MB)
Bartender	1	5.15	0.76	0.37	6.28
	2	5.72	1.35	0.37	7.44
	3	6.14	2.77	0.37	9.28
	4	6.28	7.33	0.37	13.98
Cinema	1	3.84	0.55	0.37	4.76
	2	4.25	0.98	0.37	5.60
	3	4.58	1.99	0.37	6.94
	4	4.67	5.32	0.37	10.36

**Table 12:** Ablation study results showing the RGB-PSNR range covered by the evaluated rate points and the corresponding BD-rate reduction.

Method	RGB-PSNR Range (dB)	BD-Rate
First-stage only	34.3–36.7	−30.9%
Two-stage VQ (no grouping)	35.1–38.1	−23.9%
Two-stage VQ + grouping	36.7–43.0	1.0%
Two-stage VQ + KNN grouping	36.9–44.1	−3.5%
Two-stage VQ + KNN grouping + zero-masking	34.6–44.1	−17.2%
Proposed (KNN grouping + zero-masking + flexible grouping)	34.4–44.1	−22.3%

**Table 13:** Average encoding and decoding times of the GSC anchor and the proposed method on the Bartender and Cinema datasets. Each result was averaged over five runs. The VQ time is included in the total time of the proposed method.

**(a) Encoding time**					
**Dataset**	**RP**	**Anchor Total (s)**	**Proposed Total (s)**	**VQ Component (s)**	**Proposed/Anchor**
Bartender	1	24.55	10.76	3.25	0.44
	2	50.53	14.55	6.83	0.29
	3	61.96	18.01	9.37	0.29
	4	68.64	25.20	13.09	0.37
Cinema	1	31.23	9.40	3.55	0.30
	2	44.76	12.48	6.53	0.28
	3	51.81	16.01	9.53	0.31
	4	58.58	22.11	12.45	0.38
**(b) Decoding time**					
**Dataset**	**RP**	**Anchor Total (s)**	**Proposed Total (s)**	**VQ Component (s)**	**Proposed/Anchor**
Bartender	1	0.68	0.98	0.42	1.44
	2	1.22	0.99	0.43	0.81
	3	1.41	1.01	0.41	0.72
	4	1.78	1.24	0.44	0.70
Cinema	1	0.82	0.76	0.37	0.93
	2	1.08	0.74	0.34	0.69
	3	1.25	0.79	0.32	0.63
	4	1.52	0.91	0.33	0.60

## Data Availability

The experiments were conducted using the MPEG GSC benchmark contents and the corresponding official experimental software and renderer. The parameter settings and rate-point configurations required to reproduce the reported results are provided in the manuscript. Additional implementation details may be obtained from the corresponding author upon reasonable request, subject to the access and distribution conditions of the MPEG GSC materials.
